# Maximizing Metabolic Synergy: A Review of Dual Incretin Therapy as a Step-Up Strategy Following Glucagon-Like Peptide-1 (GLP-1) Monotherapy Failure or Optimization

**DOI:** 10.7759/cureus.112694

**Published:** 2026-07-15

**Authors:** Soroush Ansari Lari, Maya S Zumot, Manar A Alrashid

**Affiliations:** 1 Department of Medicine, Royal College of Surgeons in Ireland - Medical University of Bahrain, Busaiteen, BHR; 2 Department of Internal Medicine, Royal Blackburn Hospital, Blackburn, GBR; 3 Department of Management, University of Liverpool, Liverpool, GBR

**Keywords:** body recomposition, dual incretin coagonism, gip/glp-1 receptor coagonists, glp-1 agonist, hfpef, incretin-based therapies, metabolic plateau, step-up therapy, tirzepatide, type 2 diabetes

## Abstract

While single-pathway glucagon-like peptide-1 (GLP-1) receptor agonists (RAs) have transformed metabolic medicine, patients encounter therapeutic ceilings, glycemic plateaus, and weight-loss stagnation despite ongoing treatment. The emergence of dual glucose-dependent insulinotropic polypeptide (GIP) and GLP-1 receptor (GLP-1R) co-agonists offers a novel multi-receptor mechanism that can overcome these single-pathway limitations. This review synthesizes current clinical evidence supporting the transition from single-pathway GLP-1 monotherapy to dual-pathway co-agonism and outlines practical, evidence-based clinical strategies to safely and effectively implement this step-up therapy. A comprehensive literature search was conducted across the PubMed, Embase, and Google Scholar databases for peer-reviewed articles published from inception through June 15, 2026, focusing on the landmark SURPASS, SURMOUNT, and SUMMIT trial programs, relevant cardiorenal post hoc analyses, emerging body recomposition trials, and real-world cohort-switching protocols. Based on this review, evidence from head-to-head and insulin-comparator trials demonstrates that dual GIP/GLP-1 co-agonism consistently overcomes metabolic plateaus that result from single-pathway therapy, producing superior glycated hemoglobin reduction, bariatric-level weight loss (up to 25.3%), and robust macrovascular and heart failure protection, including a 38% reduction in composite cardiovascular risk among patients with heart failure with preserved ejection fraction (HFpEF). Furthermore, recent phase 2 data demonstrate that the lean mass loss associated with profound weight loss can be pharmacologically managed; when tirzepatide is paired with the anti-myostatin agent apitegromab, sarcopenic risk is mitigated by nearly halving lean mass loss compared with dual-incretin monotherapy (14.6% vs. 30.2%), though whether this muscle-sparing synergy extends to single-pathway GLP-1 agents remains unverified. The transition from single-pathway GLP-1R agonism to dual incretin therapy reflects a broader shift toward comprehensive metabolic risk reduction rather than glycemic control alone. To safely maximize therapeutic benefits while maintaining tolerability in patients with treatment plateaus, clinicians should use structured escalation protocols characterized by clear candidate phenotyping, direct next-dose conversion without prolonged washout periods, and conservative dose-reset titration schedules to proactively mitigate gastrointestinal adverse events.

## Introduction and background

The global convergence of type 2 diabetes mellitus (T2DM) and obesity is presenting an increasingly difficult challenge to metabolic medicine, requiring highly effective, durable therapeutic interventions [1]. This clinical challenge is underscored by alarming epidemiologic data; as of 2022, 43% of the global adult population was overweight, marking a profound increase from 25% in 1990 [2]. Crucially, this includes over 890 million adults living with obesity, which is clinically defined as a body mass index (BMI) of ≥30 kg/m². This means that approximately 16% of the world's adult population now meets the criteria for obesity, reflecting a worldwide prevalence that more than doubled between 1990 and 2022 [2]. To address this compounding public health crisis, the metabolic treatment paradigm has shifted dramatically. In recent years, glucagon-like peptide-1 (GLP-1) receptor agonists (RAs) have revolutionized the landscape and broadened treatment options [1]. By mimicking native incretin hormones to augment glucose-dependent insulin secretion, delay gastric emptying, and suppress appetite, GLP-1 RA monotherapy has gained popularity and has demonstrated excellent efficacy in achieving glycemic control and significant weight reduction [1,3].

Despite their success, GLP-1 RA monotherapy has clear clinical limitations [3]. In routine practice, a significant cohort of patients receiving optimal or maximally tolerated doses of GLP-1 RAs (semaglutide 2.4 mg weekly or liraglutide 3.0 mg daily [1]) will eventually reach a therapeutic plateau [3,4]. For these patients, therapeutic progress may stall, delaying the achievement of optimal glycemic control and weight-loss targets. Although treatment intensification may be needed, escalation to insulin or complex multidrug regimens can increase the treatment burden, introducing issues such as treatment adherence [1]. This common clinical challenge highlights the limitations of single-receptor agonism and underscores the need for therapeutic strategies capable of providing additional metabolic benefit [3].

In current medical practice, a therapeutic plateau is defined as a patient's failure to achieve further meaningful improvements in glycemic control or weight loss despite three to six months of adherence to maximally tolerated or approved maintenance doses of GLP-1 RAs, such as semaglutide 2.4 mg weekly or liraglutide 3.0 mg daily [1]. In standard metabolic medicine, this optimal baseline is not determined through routine pharmacokinetic testing because engineered structural modifications, such as fatty acid conjugation or fusion to IgG-Fc regions, endow these peptide analogs with prolonged, highly predictable half-lives and minimal renal clearance, rendering routine plasma concentration monitoring clinically unnecessary [5]. Instead, the maximum dose is defined in two ways: by the fixed, regulatory-approved maintenance ceilings derived from clinical trials and by the maximally tolerated dose, the highest escalation step an individual can sustain before prolonged drug exposure triggers dose-limiting gastrointestinal toxicity [5]. This clinical plateau is driven by a therapeutic ceiling, the pharmacologic point at which a drug's maximum biologic efficacy is achieved because its targeted single-receptor pathway has become fully saturated or desensitized, meaning further dose escalation yields no additional metabolic benefit [1,3]. Establishing this definitive baseline is crucial to differentiate true, pathway-mediated ceiling effects from premature treatment discontinuation, variable patient adherence, or suboptimal drug titration [1].

Allowing a patient to remain at a single-pathway therapeutic ceiling carries severe physiologic consequences; prolonged periods of even mild hyperglycemia or sustained visceral adiposity can lead to irreversible microvascular and macrovascular damage due to the phenomenon of metabolic memory, whereby early suboptimal metabolic control continuously drives diabetic complications later in life [6]. Beyond these vascular risks, stalling at a therapeutic plateau exerts a profound psychological toll on patients, frequently leading to treatment burnout, decreased adherence, and a regression to poor dietary habits [1]. When clinicians fail to escalate treatment beyond this single-pathway ceiling despite unmet goals, this results in a state of clinical inertia, directly compounding long-term patient morbidity, mortality, and progressive disease complications [1,3,6].

To overcome these limitations, attention has increasingly shifted toward dual incretin co-agonism, which simultaneously targets both the GLP-1 and glucose-dependent insulinotropic polypeptide (GIP) receptors [3]. While GIP activity was historically considered too impaired to be useful in metabolic disease, contemporary research demonstrates that co-activation of both pathways produces a powerful synergistic effect across complementary metabolic nodes in the pancreas, brain, and white adipose tissue [3]. This step-up strategy is robustly supported by the SURPASS and SURMOUNT clinical trial programs evaluating the dual agonist tirzepatide, which consistently demonstrate that dual agonism outperforms traditional GLP-1 RA monotherapy in reducing glycated hemoglobin (HbA1c) and achieving significant weight loss [4,7].

The aim of this review is to evaluate the physiologic rationale for transitioning from single to dual incretin stimulation, synthesize the clinical trial data supporting this transition, and outline practical clinical protocols for safely stepping up patients who have reached a therapeutic ceiling on standard GLP-1 RA monotherapy.

## Review

Methods

Search Strategy and Information Sources

A systematic literature search was conducted using PubMed, Embase, and Google Scholar to identify relevant clinical trials and peer-reviewed studies published from database inception through June 15, 2026. The search strategy combined terms related to dual incretin therapy, active comparators, metabolic outcomes, and body composition. The main Boolean search string was: ("tirzepatide" OR "dual incretin" OR "GIP/GLP-1 co-agonism" OR "LY3298176") AND ("semaglutide" OR "GLP-1 receptor agonist" OR "active comparator" OR "insulin") AND ("metabolic plateau" OR "step-up therapy" OR "cohort-switching" OR "secondary intensification") AND ("myostatin inhibition" OR "activin receptor type II" OR "apitegromab" OR "bimagrumab" OR "trevogrumab" OR "enobosarm" OR "body composition" OR "lean mass preservation").

No language restrictions were applied. Reference lists of relevant articles were also screened to identify additional studies that met the eligibility criteria.

Inclusion and Exclusion Criteria

Studies were included if they were multicenter phase 2 or phase 3 randomized controlled trials, long-term open-label extension studies, or prospective real-world cohort-switching studies. Eligible studies focused on dual GIP/GLP-1 receptor (GLP-1R) co-agonism, particularly trials from the SURPASS program in type 2 diabetes, the SURMOUNT program in chronic weight management and obesity-related comorbidities, and the SUMMIT trial in HFpEF and obesity.

Studies were also considered eligible if they examined emerging combination strategies involving dual incretin therapy with body composition-modifying agents, including anti-myostatin monoclonal antibodies, dual anti-activin/myostatin therapies, or selective androgen receptor modulators.

Studies were excluded if they were narrative reviews, case series, preclinical animal studies, or phase 1 single-dose pharmacokinetic studies. Priority was given to peer-reviewed publications reporting direct head-to-head active-comparator outcomes, insulin-comparator dose-escalation algorithms, post hoc cardiorenal outcomes, or dual-energy X-ray absorptiometry-derived body composition measures.

Data Extraction and Statistical Framework

Data were extracted using a structured approach to ensure consistency across studies. Key outcomes included mean percentage change in body weight, absolute change in HbA1c, cardiometabolic outcomes, safety findings, and body composition measures where available.

Where reported, outcomes were classified according to the original trial estimands. The treatment regimen estimand was interpreted as an intention-to-treat approach that included all randomized participants regardless of treatment discontinuation or rescue therapy. The efficacy estimand was interpreted as the effect observed while participants remained on the assigned treatment without rescue therapy.

Given the heterogeneity in study design, populations, comparators, treatment duration, and outcome reporting, a formal meta-analysis was not performed. Instead, findings were summarized narratively and compared across trial programs according to therapeutic indication, comparator type, metabolic outcomes, and body composition endpoints.

Pathophysiological rationale: from single to dual incretin agonism

Limitations of Single-Pathway Stimulation and the GLP-1 RA Ceiling Effect

GLP-1 RAs exert their metabolic effects by binding to the G-protein-coupled GLP-1Rs, which are predominantly expressed in pancreatic β-cells, central nervous system (CNS) satiety centers, and the gastrointestinal tract [3]. Once activated, GLP-1R triggers a signaling cascade via the stimulatory G-protein subunit (Gαs), activating adenylate cyclase to increase intracellular cyclic adenosine monophosphate (cAMP) and protein kinase A (PKA) [8].

This intracellular signaling cascade serves as the immediate trigger for vital downstream, organ-specific homeostatic processes [3]. Within pancreatic β-cells, elevated cAMP acts through both PKA and exchange protein directly activated by cAMP 2 (Epac2) to prime the exocytotic machinery, augmenting glucose-dependent insulin secretion [3]. Beyond immediate exocytosis, this downstream pathway promotes long-term insulin biosynthesis by activating pancreatic and duodenal homeobox 1 (Pdx1), a master transcription factor that binds to the insulin promoter to upregulate its expression [3]. Simultaneously, GLP-1 coordinates blood glucose reduction by suppressing glucagon release from neighboring α-cells. This effect is mediated through an indirect, localized paracrine network within the pancreatic islets, where the downstream cascade stimulates the release of insulin, zinc, gamma-aminobutyric acid (GABA), amylin, and somatostatin to suppress glucagon secretion and reduce hepatic glucose production [3]. Outside the endocrine pancreas, this cascade acts on CNS satiety centers to diminish both homeostatic and hedonic food intake [3]. Concurrently, it targets the gastrointestinal tract to reduce smooth muscle motility and delay gastric emptying, thereby slowing postprandial macronutrient absorption and stabilizing metabolic fluctuations [3].

While highly effective at therapeutic onset, chronic hyperstimulation of this single-receptor pathway activates homeostatic counterregulatory mechanisms [3,8]. Prolonged GLP-1R occupancy leads to phosphorylation by G-protein-coupled receptor kinases (GRKs), which recruit β-arrestin-1 and β-arrestin-2 proteins [8]. This process induces desensitization, clathrin-dependent receptor endocytosis, and downregulation of surface GLP-1Rs [8]. Consequently, this leads to the ceiling effect, or therapeutic plateau, in which escalating doses yield minimal additional metabolic benefit or satiety but significantly amplify gastrointestinal adverse effects through localized emetic pathways in the area postrema [3].

The Evolving Role of Glucose-Dependent Insulinotropic Polypeptide

Historically, the physiologic relevance of GIP in the management of T2DM was discounted [3]. Early studies demonstrated that, while healthy individuals have a robust insulinotropic response to native GIP, patients with T2DM exhibit a blunted pancreatic response. This finding led to the assumption that the GIP pathway is irreversibly impaired [3,8].

More recently, molecular pharmacology has overturned this narrative by demonstrating that the therapeutic potential of GIP is not lost; rather, it depends on the underlying metabolic environment [3]. Re-establishing near-euglycemia through single-agent GLP-1 therapy or other insulin-sensitizing interventions restores the insulinotropic capacity of the GIP receptor (GIPR) on β-cells [1,3]. Furthermore, even when its isolated pancreatic effect is reduced, GIP acts as a powerful metabolic regulator across extrapancreatic tissues, functioning synergistically rather than redundantly when coactivated with GLP-1 [3,8].

GIP-Mediated Adipose Tissue Remodeling and Central Satiety Pathways

Unlike GLP-1R, which is sparsely expressed in adipocytes, GIPR is highly expressed in white adipose tissue and acts as a dynamic coordinator of lipid-buffering systems [3,8]. In the postprandial state, GIP increases blood flow to adipose tissue, enhances endothelial lipoprotein lipase (LPL) activity, and promotes efficient lipid clearance in subcutaneous adipocytes [3]. Critically, chronic dual agonism induces physiologic remodeling of adipose depots [3,8]. Rather than driving pathologic hypertrophic adiposity, which results in macrophage infiltration, hypoxia, and systemic insulin resistance, GIPR activation favors healthy hyperplastic adiposity [3]. This expansion of healthy subcutaneous fat depots buffers lipotoxicity, decreases ectopic fat deposition in visceral organs, skeletal muscle, and the liver, and markedly downregulates circulating free fatty acids (FFAs) and systemic proinflammatory cytokines [3,8]. Within the CNS, GIPR is highly expressed in distinct nuclei of the hypothalamus and hindbrain, including the arcuate nucleus and the nucleus tractus solitarius [3]. Coactivation of GIPR signaling directly complements GLP-1 pathways by reinforcing central anorexigenic signaling, modulating hedonic food reward processing, and mitigating the acute nausea pathways activated by high-dose GLP-1R hyperstimulation [3,8].

Synergistic Metabolic Mechanisms of Dual Co-agonism

Administration of a single unimolecular co-agonist capable of binding both GLP-1R and GIPR unlocks a synergy that cannot be replicated by escalating a single pathway [3]. In the pancreas, concurrent Gαs coupling from both receptors creates a cooperative intracellular cAMP pool, augmenting glucose-dependent insulin secretion by enhancing insulin granule exocytosis through both PKA- and Epac2-mediated pathways [8]. Simultaneously, the complementary extrapancreatic actions of the two hormones synchronize: GLP-1 slows gastric motility and suppresses glucagon release from α-cells, while GIP increases nutrient-induced insulin secretion and optimizes lipid disposal in peripheral adipose tissue without causing fasting hypoglycemia [3,8]. This multi-tissue crosstalk allows dual co-agonists to overcome the conventional biologic plateaus of single-agent monotherapy, delivering improved glycemic control and metabolic optimization at doses that avoid dose-limiting gastrointestinal toxicity [3,4,7].

Clinical evidence: breaking through the plateau

Glycemic Superiority: Head-to-Head Glycated Hemoglobin Clearance Across the SURPASS Program

To establish the clinical validity of stepping up from monotherapy to dual-pathway incretin agonism, the SURPASS clinical trial program [9] provides the most robust randomized controlled trial evidence. The efficacy of dual GLP-1/GIP co-agonism was initially demonstrated in the SURPASS-1 trial, which evaluated the drug as strict monotherapy without concurrent background diabetes medications [9]. Adults diagnosed with early-stage T2DM that was poorly controlled by diet and exercise alone were monitored under an efficacy estimand framework (evaluating on-treatment effects without the confounding influence of rescue therapies). From an overall study baseline HbA1c of 7.94%, participants achieved marked dose-dependent glycemic clearance. At the maximum 15 mg dose, tirzepatide induced a least-squares mean HbA1c reduction of 2.07% (standard error (SE) 0.10%), translating to an estimated treatment difference vs. placebo of -2.11%. Furthermore, 102 participants (88%) in this maximum-dose cohort successfully reached the therapeutic target of HbA1c <7.0% (with a range of 87-92% across all active treatment arms vs. 19% for placebo). This study demonstrated that dual agonism possesses a high intrinsic baseline capacity for glycemic clearance, free from the confounding or additive effects of concurrent oral antihyperglycemic medications.

The pivotal comparison was made in the SURPASS-2 trial [4], which evaluated the efficacy of the dual co-agonist tirzepatide directly against the highest standard dose of single-pathway GLP-1 RA monotherapy, semaglutide 1.0 mg weekly, over a 40-week period in patients with T2DM. Mainstream efficacy data analyzed under the treatment-regimen estimand framework (modified intention-to-treat analysis) revealed that tirzepatide, at its maximum dose, significantly outperformed semaglutide monotherapy in mean HbA1c reductions, yielding decreases of 2.30 percentage points and 1.86 percentage points, respectively [4]. This resulted in an estimated mean treatment difference of -0.45 percentage points, confirming both noninferiority and superiority [4]. Moreover, from a therapeutic optimization standpoint, 82% to 86% of patients receiving the dual co-agonist successfully met the American Diabetes Association (ADA) target of HbA1c <7.0% (compared with 79% in the semaglutide group) [4]. Furthermore, strict normoglycemia (HbA1c <5.7%) was achieved by 27% to 46% of participants in the dual-agonist arms, peaking at 46% in the 15 mg tier relative to just 19% of those assigned to semaglutide monotherapy [4]. This dramatic glycemic clearance is driven by the synergistic pancreatic effects detailed in the pathophysiological rationale; the simultaneous activation of GIP and GLP-1Rs restores β-cell glucose sensitivity and insulin secretory capacity to a degree that single-receptor hyperstimulation cannot match.

To evaluate whether dual incretin therapy could serve as an effective alternative to intensive, daily injectable regimens, the SURPASS-3 trial [10] compared once-weekly dual co-agonism with once-daily titrated insulin degludec over 52 weeks. Insulin degludec represents an ultra-long-acting basal insulin engineered to minimize glycemic variability. Despite intensive treat-to-target basal titration, the dual incretin therapy cohort receiving the maximum dose of 15 mg achieved a significantly greater mean reduction in HbA1c (2.37% vs. 1.34%) [10]. More importantly for clinical optimization, a continuous glucose monitoring (CGM) substudy within SURPASS-3 revealed that patients receiving 15 mg of dual incretin therapy spent an average of 140 minutes more per day within the target glycemic range (70-140 mg/dL) than the degludec cohort, without increasing their risk of nocturnal or severe hypoglycemia [10].

When patients progress to more advanced disease requiring insulin-based therapy, the clinical benefits of dual incretin agonism become increasingly apparent. The SURPASS-5 trial [11] demonstrated that the addition of tirzepatide to basal insulin glargine significantly improved glycemic control in individuals with inadequately controlled T2DM. At week 40, patients receiving tirzepatide 15 mg achieved a mean HbA1c reduction of 2.34% from baseline, compared with 0.86% in the placebo group [11]. Furthermore, 85-90% of participants treated with tirzepatide attained the clinically recommended HbA1c target of <7.0%, with the 15 mg dose achieving the upper limit of 90% [11].

The clinical utility of stepping up to dual-pathway therapy was further underscored in the SURPASS-6 trial [12], which evaluated intensification of insulin glargine by comparing the addition of once-weekly tirzepatide with a standard basal-bolus regimen using thrice-daily prandial insulin lispro [12]. At week 52, the pooled tirzepatide cohort achieved a highly significant mean HbA1c reduction of 2.1% from a baseline of 8.8%, compared with a reduction of just 1.1% in the insulin lispro arm, bringing the final mean HbA1c levels down to 6.7% and 7.7%, respectively [12]. Furthermore, 68% of the tirzepatide group successfully reached the therapeutic target of <7.0%, compared with 36% of the insulin lispro cohort [12]. Beyond superior glycemic clearance, the benefits of dual co-agonism over insulin intensification were evident in body composition and safety endpoints: tirzepatide-treated patients achieved an estimated mean weight loss of 9.0 kg, whereas the insulin lispro arm experienced a mean weight gain of 3.2 kg (an estimated treatment difference of -12.2 kg) [12]. Crucially, this metabolic optimization occurred alongside a dramatic reduction in hypoglycemia risk, with event rates of 0.4 events per patient-year in the pooled tirzepatide cohort versus 4.4 events per patient-year with insulin lispro [12].

While the head-to-head data establish the clear superiority of dual co-agonism over standard GLP-1 monotherapy, it is equally important to evaluate how this strategy fits into broader clinical escalation pathways. In clinical practice, when single-pathway GLP-1 RA therapy stalls or fails to maintain glycemic control, traditional therapeutic guidelines typically direct clinicians to add advanced injectable options (basal insulins such as degludec or glargine) to the patient's regimen. However, this conventional approach often introduces the well-recognized adverse effects of weight gain and an increased risk of hypoglycemia. The broader SURPASS clinical framework, particularly SURPASS-3 and SURPASS-5, directly challenged this paradigm by demonstrating that switching a plateaued patient to dual incretin therapy, rather than adding traditional basal insulin, yields substantially superior clinical outcomes. By introducing the second incretin pathway, GIP, alongside GLP-1, this switch strategy improves glycemic control more effectively while helping prevent the weight gain and glycemic variability characteristically associated with insulin initiation.

Bariatric-Level Weight Attrition: Breaking the Mass Plateau

While glycemic optimization is foundational, the therapeutic ceiling is most frequently encountered by clinicians and patients during weight management therapy [7]. Single-pathway GLP-1 RAs typically produce a weight-loss plateau at approximately 36-48 weeks due to CNS adaptations and chronic counterregulatory receptor desensitization [3]. The landmark SURMOUNT-1 trial illustrated how dual GLP-1/GIP co-agonism completely rewrites this metabolic trajectory in adults with obesity [7]. Over a 72-week treatment duration, participants receiving the maximum 15 mg dose of the dual co-agonist achieved an unprecedented average body weight reduction of 20.9% (equivalent to a mean loss of approximately 22 kg), compared with a 3.1% reduction in the placebo cohort [7]. Crucially, this step-up strategy demonstrated surgical-level efficacy distributions, with 50% and 57% of participants in the 10 mg and 15 mg dual-agonist arms, respectively, successfully achieving 20% or more total body weight loss from baseline, compared with a mere 3% in the placebo group.

Moreover, the SURMOUNT-2 trial evaluated weight management specifically in a T2DM population, demonstrating a mean weight loss of 14.7% with the dual agent [13]. Because patients with concurrent T2DM have historically been highly resistant to standard anti-obesity pharmacotherapies, achieving double-digit weight reduction represents a major clinical breakthrough [13]. This level of adipose tissue reduction approaches the efficacy thresholds traditionally seen only with metabolic or bariatric surgery, such as laparoscopic sleeve gastrectomy, establishing dual-pathway therapies as a viable nonsurgical alternative for substantial weight loss [8,13].

To further test whether dual co-agonism could induce weight loss even after a patient had maximized nonpharmacological interventions, the SURMOUNT-3 trial randomized individuals who had already achieved a ≥5.0% reduction in body weight through a rigorous 12-week intensive lifestyle modification program [14]. Upon entering the 72-week double-blind phase, patients randomized to maximally tolerated doses of the dual incretin achieved an additional mean weight reduction of 18.4%, whereas the placebo group experienced a weight gain of 2.5% [14]. When evaluating the total cumulative journey from the start of the lifestyle lead-in through the end of the trial, the tirzepatide sequence produced a total weight change of -24.3% compared with just -4.5% in the lifestyle-then-placebo cohort (with efficacy estimates of -26.6% vs. -3.8%, respectively) [14]. This underscores that dual incretin therapy not only complements behavioral modifications but also independently engages additional physiological pathways to reduce adipose tissue when traditional metabolic systems plateau [3,14].

The chronic nature of this metabolic ceiling and the need for continuous, multi-receptor stimulation were further explored in the SURMOUNT-4 maintenance trial [15]. In this study, participants with overweight or obesity received open-label dual co-agonist therapy for an initial 36 weeks, achieving a substantial mean weight loss of 20.9% [15]. Those subsequently randomized to continue dual incretin therapy for an additional 52 weeks achieved a further mean weight change of -5.5% (with efficacy estimates of -6.7%), bringing their total cumulative 88-week weight loss from week 0 to week 88 to 25.3% [15]. Conversely, patients who were withdrawn from the drug and switched to placebo experienced a rapid reversal of metabolic suppression, regaining 14.0% of their body weight from week 36 to week 88, leaving them with an overall baseline weight reduction of 9.9% at the end of the trial [15]. Crucially, 89.5% of participants who continued dual incretin therapy maintained at least 80% of the weight lost during the 36-week lead-in period, compared with 16.6% in the placebo cohort [15].

Post hoc analyses of the SURMOUNT-4 trial demonstrated that drug withdrawal and subsequent weight regain were inextricably coupled with a rebound and worsening of systolic blood pressure, fasting insulin, and circulating HbA1c levels [15]. This trial provides strong clinical evidence that the therapeutic plateau is a state of active biological counterregulation; single-pathway stagnation requires long-term optimization with dual-pathway therapies rather than transient treatment cycling [3,15]. By leveraging GIP-mediated subcutaneous adipose tissue remodeling and central neurometabolic satiety cross-talk, dual incretin therapy effectively circumvents the metabolic deceleration and hunger surges that normally contribute to the single-pathway GLP-1 RA weight-loss plateau [3].

Systemic Cardiometabolic Optimization: Cardiovascular and Macrovascular Protection

Transitioning a patient from single to dual incretin therapy marks a fundamental shift from simple biomarker modification to a strategy that stimulates multiple pathways. Foundational evidence for cardiovascular safety initially emerged from the open-label, parallel-group SURPASS-4 trial, which was designed to demonstrate cardiovascular noninferiority rather than superiority over insulin glargine [16]. In patients with long-standing T2DM and established cardiovascular disease or high cardiovascular risk, pooled tirzepatide treatment demonstrated a numerical 26% reduction in major adverse cardiovascular events major adverse cardiovascular events (MACE-4; a composite of cardiovascular death, myocardial infarction, stroke, and hospitalization for unstable angina) compared with daily titrated glargine [16]. While this macrovascular separation did not achieve formal statistical significance because the confidence interval crossed unity, the trial successfully established the requisite safety profile [16]. At 52 weeks, this macrovascular safety was accompanied by superior glycemic efficacy, as tirzepatide achieved a mean HbA1c reduction of 2.43% with the 10 mg dose and 2.58% with the 15 mg dose, compared with 1.44% with glargine (achieving an estimated treatment difference of up to 1.14%) [16]. Crucially, this robust glycemic control was achieved with a significantly lower incidence of hypoglycemia (6-9% vs. 19% with glargine), particularly among participants not receiving background sulfonylureas, in whom event rates dropped to 1-3% [16].

The clinical evidence for macrovascular protection was highlighted by the multicenter, double-blind SURPASS-CVOT trial [17]. As the first active-comparator trial of its kind, it directly compared the dual GLP-1/GIP agonist tirzepatide (up to 15 mg weekly) with a highly effective single-pathway GLP-1 RA, dulaglutide (1.5 mg weekly), in a modified intention-to-treat population of 13,165 patients with T2DM and atherosclerotic cardiovascular disease (ASCVD) (6,586 patients in the tirzepatide group and 6,579 in the dulaglutide group) [17]. Ultimately, tirzepatide met its primary endpoint, demonstrating noninferiority for the three-component MACE composite of death from cardiovascular causes, myocardial infarction, or stroke [17]. Crucially, from an optimization perspective, a primary MACE event occurred in 12.2% (801) of patients in the tirzepatide cohort compared with 13.1% (862) of those treated with dulaglutide [17]. Alongside this proven cardiovascular safety profile, patients in the dual-agonist cohort experienced a greater reduction in body weight from baseline than those treated with dulaglutide (11.6% vs. 4.5%), accompanied by greater reductions in HbA1c (1.66% vs. 0.88%) and a more profound decrease in fasting triglyceride levels [17]. Furthermore, all-cause mortality was significantly lower with tirzepatide, largely driven by a reduction in noncardiovascular deaths [17]. These findings suggest that the superior weight-loss and lipid-lowering effects inherent to GIP/GLP-1 synergy may translate into a lower overall systemic risk burden [3,17].

A subsequent broad six-component post hoc analysis of the SURPASS-CVOT trial further demonstrated that dual therapy resulted in a substantial 16% reduction in a combined cardiorenal composite endpoint comprising all-cause mortality, myocardial infarction, stroke, coronary revascularization, heart failure hospitalization, and adverse kidney outcomes compared with single-receptor agonism [18]. Specifically, among the 13,165 patients evaluated over a median treatment duration of 46.9 months, this primary composite cardiorenal endpoint occurred in 23.7% (1,559) of tirzepatide-treated patients compared with 27.4% (1,803) of the dulaglutide cohort [18]. The durability of this multireceptor advantage was further supported by sensitivity analyses, which demonstrated sustained clinical benefit when the endpoint was narrowed to either a five-component configuration excluding kidney outcomes or a traditional four-component macrovascular composite excluding both renal and heart failure events [18].

Complementing the macrovascular protection observed in atherosclerotic cohorts, the landmark SUMMIT trial explored the use of dual incretin therapy in 731 patients with heart failure with preserved ejection fraction (HFpEF) and concurrent obesity [19]. HFpEF represents a highly complex metabolic-cardiovascular phenotype in which mechanical adipose tissue pressure and chronic inflammation contribute to myocardial stiffness. In this international, double-blind trial, 364 participants were assigned to tirzepatide and 367 to placebo. Treatment with the dual co-agonist achieved a 38% reduction in the combined primary risk of cardiovascular death or worsening heart failure outcomes compared with placebo (9.9% in the tirzepatide cohort vs. 15.3% in the placebo group), driven primarily by a 46% reduction in worsening heart failure events [19]. Additionally, dual incretin therapy met its coprimary endpoint by improving patient-reported health status, achieving a mean change from baseline to 52 weeks in the Kansas City Cardiomyopathy Questionnaire Clinical Summary Score (KCCQ-CSS) of 19.5 points compared with 12.7 points in the placebo group, alongside an 18.3 m increase in the six-minute walk distance and a 38.8% reduction in high-sensitivity C-reactive protein (hsCRP) [19]. These robust cardioprotective data suggest that remaining on standard single-pathway therapy when an effective step-up option is available may leave patients unnecessarily vulnerable to heart failure progression and systemic vascular complications [1,17,19].

The Body Recomposition Dilemma: Sarcopenic Risk and Lean Mass Preservation

As incretin-based therapeutics achieve unprecedented weight-loss rates, a major clinical challenge has emerged regarding the preservation of lean body mass during rapid weight loss [3]. Standard calorie-restricted weight-loss interventions and early-generation GLP-1 RA monotherapies typically result in a distinct body composition penalty, in which a substantial portion of the total weight lost is derived from lean skeletal muscle rather than fat stores [7]. This structural depletion raises long-term concerns about frailty, reduced functional exercise tolerance, and a reduced resting metabolic rate, which can inadvertently accelerate a metabolic plateau.

While the magnitude and speed of weight reduction driven by GIP/GLP-1 receptor (GLP-1R) co-agonists could theoretically increase the absolute loss of lean mass, the physiological integration of GIP signaling introduces a unique tissue-level advantage [3,8]. Unlike single GLP-1 RAs, GIPR activation directly modulates subcutaneous adipose tissue blood flow and enhances lipid storage flexibility, potentially promoting preferential fat oxidation while sparing vital structural proteins when combined with protective clinical strategies.

To counteract this sarcopenic risk, metabolic research is shifting toward combination pharmacology designed to uncouple lean mass decline from total fat loss [8,20]. The landmark phase 2 randomized controlled EMBRAZE trial demonstrated proof of concept for this approach by evaluating the highly selective anti-myostatin monoclonal antibody apitegromab, administered as an adjunctive intravenous infusion of 10 mg/kg every four weeks alongside weekly tirzepatide over 24 weeks [20]. The trial data revealed that, while the placebo plus tirzepatide group lost 30.2% of their total weight as lean mass (corresponding to a 3.5 kg reduction in lean mass), the addition of apitegromab preserved a significant portion of skeletal muscle, reducing lean mass loss to 14.6% of total weight loss (corresponding to a 1.6 kg reduction in lean mass) [20]. This resulted in a 54.9% relative preservation of lean mass (an average of 1.9 kg less lean mass loss) compared with the placebo group. Furthermore, this preservation altered the overall composition of weight loss; fat mass loss comprised a substantially larger proportion of total body weight reduction among participants who received apitegromab than among those who received placebo (85.3% vs. 69.5%), improving the overall quality of weight loss without blunting the drug's fat-reducing effects [20].

Until these combination myostatin inhibitors complete phase 3 validation, the clinical responsibility for body recomposition during a step-up protocol relies primarily on structured lifestyle intervention. Reflecting this, current protocols from ongoing clinical trials recommend pairing dual incretin stimulation with progressive resistance training and a target protein intake of 1.6 g/kg/day [21]. Preserving skeletal muscle integrity ensures that stepping up a patient does not merely lower the number on the scale but also promotes healthspan-optimizing metabolic remodeling [21].

Practical clinical strategies for step-up therapy

Phenotyping the Ideal Candidate: Defining Thresholds for Escalation

**Figure 1:**
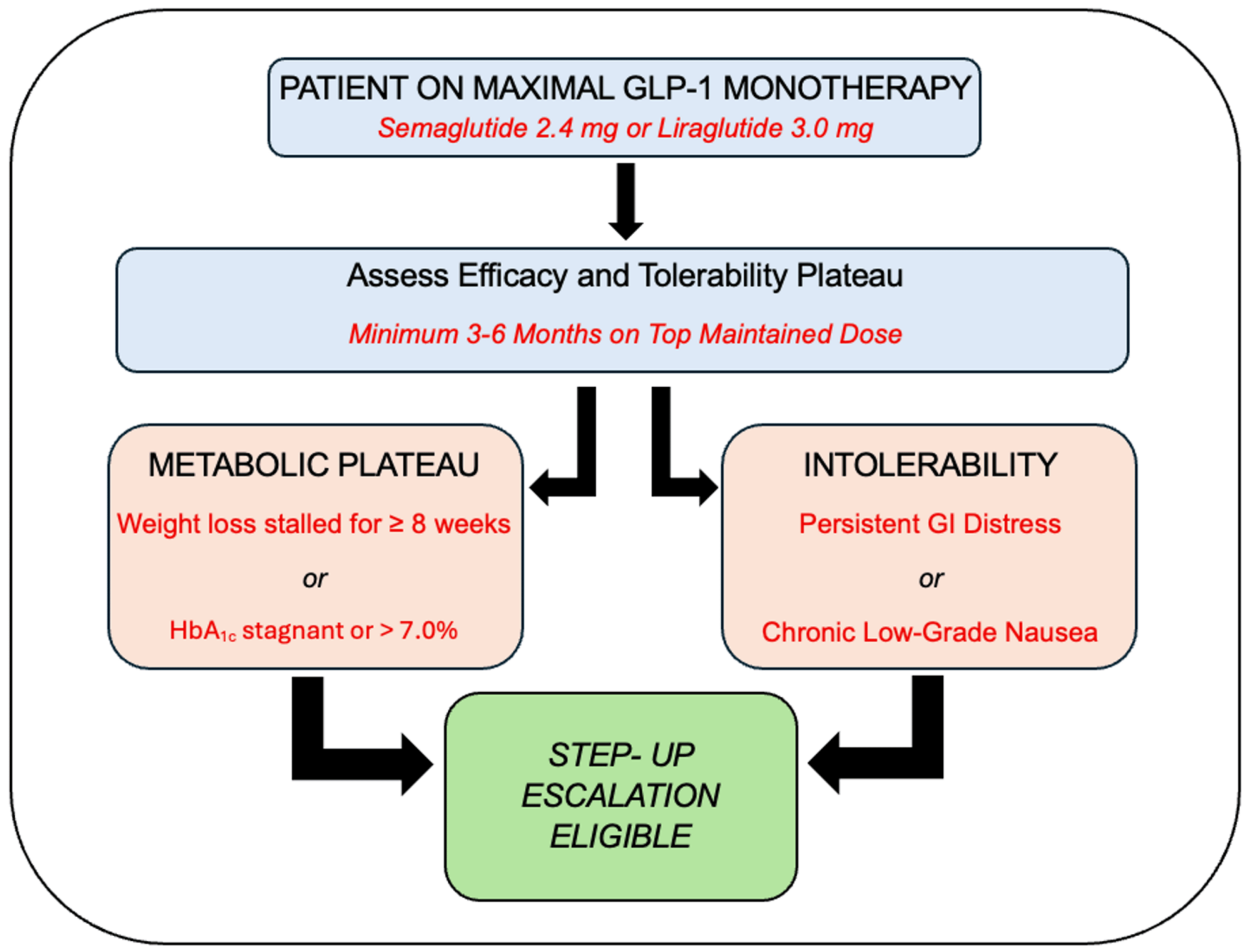
Proposed clinical phenotyping framework for incretin escalation. GLP-1, glucagon-like peptide-1; HbA1c, glycated hemoglobin; GI, gastrointestinal

Transitioning a patient from single-receptor GLP-1 monotherapy to dual GLP-1/GIP co-agonism should not be done arbitrarily; rather, it requires careful clinical phenotyping to identify those who will benefit most. Based on the outline in Figure 1, the ideal candidate for a step-up protocol meets one or more of the following clinical criteria.

Receptor-mediated weight plateau: Patients who have reached a definitive weight-loss plateau (failing to lose >1.0% of body weight over a consecutive eight-week period) despite a minimum of three to six months of adherence to the maximum FDA-approved maintenance dose of a single GLP-1 RA (e.g., semaglutide 2.4 mg weekly) [1,3].

Suboptimal glycemic control: Individuals with T2DM who fail to achieve or maintain their target HbA1c (<7.0%) or who exhibit high glycemic variability on continuous CGM despite reaching the maximally tolerated dose of standard GLP-1 RA therapy [1,4].

Primary or secondary monotherapy nonresponse: Patients exhibiting an inadequate initial metabolic response to single-pathway monotherapy, often characterized by persistent "food noise," recurrent cravings, or an inability to achieve metabolic optimization because of inherent genetic variation in single-receptor pathway sensitivity [3].

The Switching Protocol: Clinical Execution and Safety Dynamics

Because large-scale, multisociety consensus algorithms for cross-class transitions are still absent from the contemporary literature, we outline a proposed clinical framework based on established pharmacokinetic profiles and real-world institutional experience.

Once a patient is deemed an appropriate candidate for escalation, clinicians must implement the transition protocol safely to preserve therapeutic momentum without unnecessary overlap of the drugs' pharmacokinetic effects. Because both semaglutide and the dual co-agonist tirzepatide have similar pharmacokinetic half-lives of approximately seven days, a direct transition can be implemented without a prolonged washout period [3].

A practical, physiologically grounded protocol involves administering the patient's final weekly single-agent GLP-1 RA injection, waiting seven days, and initiating the dual co-agonist on the next regularly scheduled injection day. This direct next-dose conversion is intended to prevent rebound hyperglycemia and protect the patient from an immediate increase in appetite or baseline food noise.

Crucially, dose conversion should not follow a direct 1:1 milligram substitution because dual-pathway co-agonists exhibit significantly greater biological potency per molecule [3,4]. Regardless of whether a patient was stable on the maximum approved dose of a single-pathway agent (such as semaglutide 2.4 mg), safety frameworks favor a conservative introductory phase to mitigate cross-class adverse effects [4].

We propose two primary entry pathways: 1. Standard reset: Initiate the dual agonist at the introductory dose of 2.5 mg weekly for the first four weeks. This functions as a nontherapeutic adjustment phase designed to acclimate the patient's gastrointestinal tract to the newly introduced GIPR signaling pathway [3]. 2. Accelerated step-up: In patients who have demonstrated long-term tolerance of high-dose single-receptor monotherapy for more than six months without gastrointestinal adverse effects, clinical practice may permit initiation at the 5.0 mg weekly dose. However, initiating treatment at higher maintenance doses (e.g., 7.5 mg or 10.0 mg) is strongly discouraged because of the unacceptably high risk of acute dysmotility and subsequent treatment discontinuation driven by multireceptor hyperstimulation [3,4].

Mitigating Gastrointestinal Sequelae: Proactive Tolerability Mapping

Managing side effects during the cross-class switch is the primary factor influencing long-term treatment adherence. Cohort data show that approximately 20% to 30% of pretreated patients experience a temporary recurrence of mild-to-moderate gastrointestinal adverse events, including transient nausea, diarrhea, dyspepsia, and delayed gastric emptying during the initial weeks of the switch [3,4].

To flatten this side-effect curve, we recommend following a strict titration schedule. Dose escalation should follow a rigid four-week stepwise interval (e.g., 2.5 mg, 5.0 mg, 7.5 mg, and 10.0 mg). If a patient experiences persistent nausea or abdominal distress during dose escalation, the protocol should mandate maintaining the patient at that specific intermediate dose tier for an additional two to four weeks rather than forcing further upward titration.

Furthermore, patients should receive comprehensive nutritional counseling at the time of the switch, emphasizing the avoidance of high-fat and highly processed foods, which exacerbate delayed gastric emptying, while reinforcing the need for portion control and adequate hydration [1]. This counseling should also emphasize adequate protein intake (1.6 g/kg/day) and progressive resistance training to preserve skeletal muscle once dual incretin therapy is initiated [21]. This proactive approach to tolerability helps patients successfully acclimate to dual-receptor therapy, supporting long-term metabolic optimization. These measures may improve the patient experience, reduce side effects, enhance treatment adherence, and ultimately optimize clinical outcomes.

Future research and the horizon

Further Escalation: Triple Incretin Receptor Agonism

While switching to dual incretin therapy provides an effective mechanism for overcoming the therapeutic plateaus of monotherapy, metabolic medicine continues to evolve rapidly beyond dual-pathway configurations. Current clinical research centers on the development of unimolecular triple RAs that combine glucagon receptor activation with the existing GLP-1 and GIP signaling cascades [8]. This triple-pathway configuration seeks to exploit a sophisticated physiological synergy: while GIP and GLP-1 optimize insulin secretion, suppress appetite, and improve peripheral insulin sensitivity, the addition of glucagon receptor agonism independently increases basal metabolic rate, accelerates whole-body energy expenditure, and directly promotes the clearance of intrahepatic lipids [3,8].

Retatrutide (LY3437943) is the leading agent within this triple-receptor class and is currently undergoing phase 3 evaluation in the TRIUMPH clinical program [22]. Phase 2 data evaluating retatrutide in adults with obesity demonstrated a mean body weight reduction of up to 24.2% at 48 weeks of treatment, with 100% of participants receiving the highest dose achieving the clinically meaningful weight-loss threshold of ≥5.0% [22]. This tri-agonist approach appears to delay or bypass the classic biological adaptations that contribute to single-receptor weight-loss plateaus, potentially establishing a new therapeutic ceiling for patients who have exhausted dual-agonist therapies [8,22].

Mapping Critical Gaps in the Literature

As metabolic pharmacotherapy transitions toward this multi-receptor future, the literature contains several critical knowledge gaps that require immediate, structured investigation. To guide future research efforts, the academic community should prioritize the following three clinical domains:

Long-term durability matrix: While the SURMOUNT-4 trial demonstrated the rapid metabolic rebound and cardiovascular deterioration that follow the withdrawal of dual-receptor stimulation [15], long-term longitudinal studies extending beyond five to 10 years are needed. It remains to be determined whether chronic multi-receptor hyperstimulation induces permanent epigenetic remodeling of satiety pathways or whether a secondary, late-stage biological ceiling develops in patients receiving lifelong maintenance therapy [22].

Precision phenotyping and biomarker mapping: In routine clinical practice, patient responses to incretin therapies vary considerably. Future molecular research should focus on identifying specific genetic polymorphisms, circulating microRNA profiles, or baseline gut microbiota signatures that accurately predict whether a patient will respond well to GLP-1 RA monotherapy or possesses an inherent physiological profile that warrants initial treatment with dual- or triple-RAs [8].

The cross-class switching void: The most immediate clinical gap remains the lack of clear, consensus-driven switching algorithms. As discussed throughout this review, existing phase 3 clinical trials primarily evaluated incretin-naive cohorts [4,7]. To improve patient safety in real-world practice, double-blind randomized controlled trials are needed to directly evaluate the safety, gastrointestinal tolerability, and initial weight-loss trajectory of transitioning heavily pretreated patients with stalled responses to monotherapy directly to escalating doses of dual RAs [23]. Resolving these implementation gaps will help transform metabolic medicine from a reactive, trial-and-error escalation strategy into a discipline of precise, proactive, and individualized metabolic care [1,8,23].

Limitations of This Study

While this review synthesizes robust clinical data, several limitations should be acknowledged. First, the foundational evidence supporting dual incretin therapy is derived primarily from large-scale, industry-sponsored phase 3 clinical trials, including the SURPASS and SURMOUNT programs. Consequently, high-quality randomized controlled trial data specifically evaluating clinical switching dynamics, titration protocols, and real-world adherence among patients with inadequate responses to single-pathway GLP-1 RAs remain limited. Second, the included observational cohorts and expert consensus guidelines exhibit minor heterogeneity in baseline patient characteristics and operational definitions of therapeutic failure. Finally, this review was restricted to peer-reviewed articles published in English, potentially excluding regional real-world datasets or localized clinical experience with multireceptor therapies.

## Conclusions

The transition from GLP-1 RA monotherapy to dual GIP/GLP-1 receptor co-agonism represents a paradigm shift in metabolic medicine, moving clinical goals from simple biomarker adjustment to comprehensive systemic protection. The landmark SURPASS and SURMOUNT clinical trial programs consistently demonstrate that multi-receptor synergy overcomes the therapeutic ceilings, metabolic plateaus, and weight-loss stagnation frequently encountered with single-pathway agents. By leveraging the physiological interaction between the GIP and GLP-1 pathways, dual therapy delivers superior glycemic control, achieves bariatric-level weight loss, and provides robust macrovascular and heart failure protection.

Furthermore, as emerging clinical evidence highlights the need to preserve lean mass during rapid weight loss, dual incretin strategies, when combined with structured lifestyle interventions or next-generation combination therapies, provide a superior framework for healthspan-optimizing body recomposition. To safely realize these benefits in real-world clinical practice, physicians should transition from a reactive approach to a proactive, structured escalation protocol. Implementing clear thresholds for step-up eligibility, standardized next-dose titration schedules, and proactive gastrointestinal symptom management can help patients navigate the class switch with minimal disruption. As the therapeutic landscape expands toward triple-receptor tri-agonists, establishing definitive, consensus-driven switching algorithms remains a critical priority to support individualized, high-impact, and long-term metabolic preservation.
